# The Future of Porcelain Work

**Published:** 1902-12

**Authors:** N. S. Jenkins

**Affiliations:** Dresden


### THE FUTURE OF PORCELAIN WORK.[1]

BY N. S. JENKINS, D.D.S., DRESDEN.

Porcelain has always been a somewhat intractable material. Its first application to artificial teeth was unpromising. Neither substance nor color became reasonably practicable for many years, but eventually manufacturers overcame all difficulties and now are

---

[1] Read before the Swedish Dental Society, Stockholm, August, 1902.

constantly presenting us with forms and shades which are as useful as they are novel. It is, however, during the last ten years that porcelain, for other purposes than artificial teeth, has become the subject of elaborate experiment, and the progress which has thus far been made is of a most encouraging nature. By means of porcelain we are now able to make restorations of the most elaborate character in the natural teeth, and also to make pivot teeth and bridges of great strength and beauty.

There are many reasons why this work is regarded with favor. Its use is possible with very little strain upon the nerves of the patient or the operator, and the results obtained leave little to be desired in appearance or usefulness; but to secure these results requires an attention to exact manipulation which leaves nothing to chance or imagination. Careless operators are quickly condemned by these methods, but the work of skilful and patient dentists may reach a perfection hitherto unknown.

For a long time it was erroneously supposed that high-fusing bodies must of necessity be stronger than low-fusing bodies. This has been a natural conclusion, since all experiments in the direction of such materials for dental purposes have been based upon, or influenced by, the methods used in the manufacture of artificial teeth. In my paper, published in the *Dental Cosmos* for February, 1902, you will have read the table of statistics which shows the superior specific gravity and the greater strength of porcelain enamel, facts upon which I need not dwell. Let me, however, explain why, in high-fusing bodies for inlays and pivot teeth, the strength and density of artificial teeth cannot be obtained. The materials of which an American tooth is composed are subjected to considerable pressure in the mould in which it is formed. This establishes a density, through bringing the particles in more immediate contact with each other, and enables the flux to unite the infusible ingredients more perfectly than is possible in any other method. To compensate for inability to exercise pressure upon material designed for inlay work was one of the greatest problems in the evolution of porcelain enamel, and the solution was found not only in exact and elaborate preparation of the body, but also in a method of fusing, which enables one to perfectly control the heat and to accurately observe the progress of the melting. These conditions are seemingly impracticable with high-fusing bodies.

The increasing use of porcelain in the future, until it occupies the chief place in dental practice, seems to me to be assured. It is so evidently superior to the old methods, in nearly every particular, that it is certain to win its way. One by one the difficulties of working porcelain bodies have been overcome, until now, instead of being a complicated art to be practised only by specialists, it has become a simple and logical process, yielding results as exact as chemistry or mathematics. It requires only the skilful hand and the trained mind of any educated dentist, who has such breadth of culture that he can frankly reverse some of the methods he has learned to apply to gold work, to produce, in his daily practice, results as useful and practical as they are immeasurably beautiful.

